# Elevated Serum C1q Levels in Children With Sepsis

**DOI:** 10.3389/fped.2021.619899

**Published:** 2021-04-26

**Authors:** Huan Li, Juanjuan Chen, Yuanhui Hu, Xin Cai, Pingan Zhang

**Affiliations:** Department of Clinical Laboratory, Renmin Hospital of Wuhan University, Wuhan, China

**Keywords:** complement C1q, procalcitonin, sepsis, children, infection

## Abstract

**Objective:** To analyze the serum complement C1q levels in children with sepsis, and explore the suggestive effect of serum C1q levels on the condition of children with sepsis.

**Methods:** The clinical and laboratory data of children with sepsis (*n* = 95) and healthy children (*n* = 71) in Renmin Hospital of Wuhan University from January 2019 to October 2019 were collected, and each index of the two groups was compared. Then we divided children with sepsis into three subgroups based on the Pediatric Critical Illness Score (PCIS): non-critical group, critical group, and extremely critical group. The serum C1q and PCT levels of the three subgroups were analyzed, and the correlation analysis was carried out between the levels of serum C1q and PCT levels as well as PCIS among children with sepsis. Finally, we analyzed the serum C1q levels of septic children infected by different pathogens.

**Results:** The serum C1q levels of children with sepsis were significantly higher than those of healthy children (median 198.4 vs. 186.2 mg/L, *P* < 0.001). In the analysis of subgroups, the serum C1q levels of non-critical group, critical group, and extremely critical group septic children were 182.80 (166.75, 195.85) mg/L, 219.90 (209.10, 246.40) mg/L and 249.95 (239.10, 272.25) mg/L, respectively, which were correlated with the severity of the disease. At the same time, we also found that serum C1q in children with sepsis was positively correlated with PCT levels (*r* = 0.5982, *P* < 0.001), and negatively correlated with PCIS score (*r* = −0.6607, *P* < 0.001). The serum C1q levels of septic children with bacterial infections, mycoplasma infections, viral infections, and co-infection were higher than those of the control group (*P* < 0.05).

**Conclusion:** The serum levels of C1q in children with sepsis were increased and related to the severity of sepsis, suggesting that C1q may be involved in the occurrence and development of sepsis, which had reference value for the preliminary diagnosis and severity classification of sepsis.

## Introduction

Sepsis is considered as a seriously life-threatening disease (1). Its latest definition was jointly released by the American Academy of Critical Care Medicine and the European Association of Critical Care Medicine in 2016 (2). It is a fatal organ dysfunction caused by the body's uncontrolled response to infection, which has a high morbidity and mortality rate (3). There are about 48.9 million sepsis cases in the world, and 11 million sepsis-related deaths in 2017, accounting for 19.7% of the total global deaths according to an article published in *Lancet* (4). Simultaneously, sepsis is the heaviest medical burden. In 2013, the cost of sepsis treatment in the United States was up to 23.7 billion US dollars, accounting for 6.2% of the total hospitalization costs, which ranked first (5). Sepsis can occur at all ages, but studies show that children and older people are the majority (6). The research object of this paper were children.

Due to the high mortality rate of sepsis, its early warning is of great significance in improving survival rate and prognosis. The currently recognized marker of sepsis is procalcitonin (PCT) (7), which is a protein whose level in plasma increases when there are severe bacterial, fungal, and parasitic infections, as well as sepsis and multiple organ failure. However, the increase of PCT level can only last for 1–4 days (8). There are limitations in the detection of PCT for patients who cannot get timely medical visit, and some other markers such as C reactive protein (CRP) (9, 10), serum amyloid protein A (SAA) (11) along with some cytokines (12) were of poor diagnostic specificity.

In recent years, studies have found that the activation of complement system is one of the important pathogenesis of sepsis (13). The complement system is the core part of the body's innate immunity. It is composed of a variety of humoral proteins, receptors and regulatory proteins. And it can induce inflammatory, resist pathogen infection, and can be used as immune effector and regulator. Sepsis can lead to excessive activation of serum complement (14). The activated products of complement can promote inflammation by recruiting and activating white blood cells (WBC), endothelial cells and platelets (PLT), which can lead to the imbalance of inflammatory response and aggravate the inflammatory damage, eventually leading to multiple organ failure or even death (15). On the other hand, when severely infected, the body will produce compensatory complement. Serum complement is closely related to the occurrence and development of sepsis. At present, there are many studies on complement in sepsis. For example, Qi Anlong reported that anti C5a monoclonal antibody may improve the prognosis of sepsis by improving the polarization of spleen mature DC and T cells, which indicates that C5a plays an important role in the immune regulation of sepsis cells (16); Many animal experiments have found that complement components can promote histone production, further leading to cell damage and multiple organ dysfunction in septic mice. At the same time, the occurrence of septic cardiomyopathy also depends on C5a, C5a receptors and histones. The neutralization of C5a with antibodies or the absence of C5aR1 prevents the appearance of extracellular histones, cell death and organ failure in sepsis. Blocking C5a with antibodies can significantly improve the survival rate of septic mice (17); In addition to C5a, other complement components such as C3a, C4, FBP have many relevant studies in sepsis (14, 18, 19). Some scholars have found that when sepsis occurs, the complement system and neutrophils are activated by pathogens and endogenous danger signals at the same time. Complement mediates the activation of neutrophils and plays an important role in the pathophysiological process of sepsis (20).

Serum complement C1q is also an important complement component. In addition to acting as a promoter of classical complement activation pathway, serum complement C1q can activate cascade reaction to clear antigen antibody complex, and also participate in the clearance of apoptotic cells and help maintain the integrity of vascular endothelial cells. When the balance of production and consumption of C1q is broken, it will induce the occurrence and development of kidney disease, atherosclerosis and central nervous system diseases, and C1q is related to aging. At the same time, it also has the function of reducing the release of pro-inflammatory cytokines and promoting the production of anti-inflammatory mediators by macrophages, dendritic cells and microglia (21). However, there is no relevant report of complement C1q in sepsis at present. This study mainly evaluates the levels of serum complement C1q in children with sepsis, and preliminary assessment of its correlation with the severity of sepsis in children.

## Materials and Methods

### Participants

A total of 166 subjects were included in this study, including 95 cases of sepsis group (age: 4.56 ± 2.00 years) and 71 cases of control group (age: 4.46 ± 2.00 years). All subjects were recruited from the Renmin Hospital of Wuhan University from January 2019 to October 2019. The disease group was further divided into non-critical group (*n* = 48), critical group (*n* = 27) and extremely critical group (*n* = 20) according to the Pediatric Critical Illness Score (PCIS). The PCIS included heart rate, blood pressure, respiratory rate, arterial partial pressure of blood oxygen, pH value, blood sodium, blood potassium, urea nitrogen/blood creatinine, hemoglobin, gastrointestinal system symptoms (bleeding from stress ulcer or intestinal paralysis). The lower the score, the worse condition of the disease. A score of lower than 70 is extremely critical, 71–80 was critical, and higher than 80 was non-critical (22). Children with sepsis in the disease group were all in line with the “Expert Consensus on the Diagnosis and Treatment of Septic Shock in Children (2015 Edition).” In this consensus, sepsis is defined as systemic inflammatory response syndrome caused by infection. The specific diagnostic criteria are as follows: fever (rectal temperature > 38.5°C) or hypothermia (rectal temperature < 35°C), tachycardia (people with hypothermia may have no tachycardia), accompanied by at least one of the following organ dysfunctions: altered consciousness, hypoxemia, increased serum lactic acid, or bounding pulse. In the sepsis group, there were six cases of bacterial infection, 26 cases of mycoplasma infection, 16 cases of virus infection, 19 cases of co-infection, and 28 cases of unknown pathogen type. Premature infants, children with congenital underlying diseases, genetic metabolic diseases in addition to primary immunodeficiency diseases that may affect the prognosis were excluded. The control group was children who had a healthy physical examination, and excluded those who had signs of infection within the first month before enrollment or had received antibacterial drugs or other non-preventive drugs for other reasons. The study was reviewed and approved by the Ethics Committee of the Renmin Hospital of Wuhan University and approved to exempt patients with informed consent.

### Data Collection

All data were collected from the Department of Clinical Laboratory, Renmin Hospital of Wuhan University. The levels of WBC, hemoglobin (Hb), PLT, PCT, CRP, SAA, pre-albumin (PA), total protein (TP), C1q, glucose (Glu) and lactate dehydrogenase (LDH) were the first laboratory test results after admission. It is understood that in laboratory tests, WBC, PLT and Hb were analyzed by Sysmex XN-9000 automatic blood cell analyzer. serum PCT was detected by Cobas 8000 e 801 automatic chemiluminescence immunoassay analyzer produced by Roche diagnostic company, and PCT > 0.1 ng/ml indicated bacterial infection. CRP and SAA were detected by H780-3 automatic specific protein analyzer produced by Xiliheng company (Shenzhen, China). Serum PA, TP, C1q, Glu and LDH were detected by Siemens ADVIA 2400 biochemical analyzer and related reagents. Among them, C1q was detected by immunoturbidimetric assay, and C1q reagent was purchased from Beijia biochemical reagent, and there was no missing value of serum C1q. And we have access to information that could identify individual participants during or after data collection.

### Statistical Analysis

Statistical analysis was performed with the SPSS software, version24.0 (IBM, Armonl, NY, USA) and GraphPad Prism 6.0 (GraphPad Software, La Jolla, CA, USA). Single sample Kolmogorov-Smirnov (K-S) method was used to test whether the data of each group conformed to the gaussian distribution. Data conforming to gaussian distribution include age and PA; non-gaussian distribution data include WBC, Hb, PLT, PCT, CRP, SAA, TP, C1q, Glu, LDH. Gaussian distribution data (age and PA) are expressed as mean ± standard deviation (mean ± SD), the comparison between the two groups (healthy control group and sepsis group) uses the independent sample *t* test, and the Pearson product-moment correlation coefficient is used to express the correlation between the two groups; non-Gaussian distribution data (WBC, Hb, PLT, PCT, CRP, SAA, TP, C1q, Glu, and LDH) are represented by median (P_25_, P_75_), Mann-Whitney *U* test is used for comparison between the two groups (healthy control group and sepsis group). The C1q and PCT levels between subgroups are compared using the Kruskal-Wallis *H* test among multiple groups, and the comparisons are made in pairs afterwards. Spearman correlation coefficient is used to express the correlation between two sets of data (C1q and PCT, C1q and PCIS). The sex distribution belongs to count data, using the χ2 test. *P* < 0.05 means the difference is statistically significant. The box plot method is used to identify outliers. The results show that there are two outliers (high value) in WBC, one outlier (high value) in Hb, three outliers (high value) in PCT, two outliers (high value) in CRP, and one outlier (high value) in LDH. For the treatment of outliers, we did two tests before and after outlier elimination, and found that the results of the two tests were not contradictory, so we didn't eliminate the outliers. Because the PCT in our subgroup analysis also had outliers, we did two tests before and after outlier elimination in subgroup analysis, and found that the results of the two tests were not contradictory, so we did not eliminate the outliers either.

## Results

### Study Population

The clinical data of all subjects are as follows (Table 1). There was no significant difference in gender and age between the two groups (*P* > 0.05). The levels of Hb and PA in sepsis group were lower than those in healthy control group (*P* < 0.05), while WBC, PLT, CRP, SAA and LDH levels in sepsis group were higher than those in healthy control group. Serum C1q (median 198.4 vs. 186.2 mg/L, *P* < 0.001) and PCT (median 1.12 vs. 0.03 ng/ml *P* < 0.001) were the same.

**Table 1:** Clinical characteristics of the participants.

	**Sepsis (*n* = 95)**	**Healthy control (*n* = 71)**	**Statistics**	***P***
Gender (Male/Female)	57/38 (1.5:1)	42/29 (1.45:1)	χ2 = 0.017	0.896
Age (years)	4.56 ± 2.00	4.46 ± 2.00	*t* = 0.297	0.767
WBC ( × 10^9^/L)	19.40 (15.71, 24.19)	7.13 (5.85, 8.23)	*u* = 10.464	<0.001
Hb (g/L)	127 (120, 133)	133 (122, 146)	*u* = −2.975	0.003
PLT ( × 10^9^/L)	287 (249, 362)	273 (234, 307)	*u* = 2.435	0.015
PCT (ng/ml)	1.12 (0.385, 1.642)	0.03 (0.02, 0.05)	*u* = 10.522	<0.001
CRP (mg/L)	43.6 (13.34, 75.71)	0.5 (0.5, 0.8)	*u* = 10.484	<0.001
SAA (mg/L)	200 (117.04, 200)	5 (5, 6.98)	*u* = 10.779	<0.001
PA (mg/L)	145.69 ± 41.37	216.81 ± 45.90	*t* = −10.456	<0.001
TP (g/L)	68.2 (65.1, 71.8)	66.6 (63.5, 70.4)	*u* = 1.663	0.096
C1q (mg/L)	198.4 (181, 239.1)	186.2 (169, 201)	*u* = 3.835	<0.001
Glu (mmol/L)	4.51 (3.86, 5.71)	4.59 (4.26, 5.13)	*u* = −0.194	0.846
LDH (U/L)	320 (287, 386)	231 (189, 294)	*u* = 7.377	<0.001
**Type of pathogen**				
Bacteria (cases)	6	-	-	-
Mycoplasma (cases)	26	-	-	-
Virus (cases)	16	-	-	-
Multiple pathogens (cases)	19	-	-	-
Pathogen unknown (cases)	28	-	-	-

*Sex belongs to count data, chi-square test is used for comparison between the two groups, χ2 is the chi-square value; Age and PA conform to Gaussian distribution, expressed as mean ± SD, independent sample t test is used for comparison between the two groups, and t value is the test statistic; WBC, Hb, PLT, PCT, CRP, SAA, TP, C1q, Glu, and LDH do not conform to Gaussian distribution and are represented by median (P_25_, P_75_). The comparison between the two groups uses Mann-Whitney U test, and u is the test statistics*.

### Expression of C1q and PCT in Children With Sepsis in Different Subgroups

**Figure 1:**
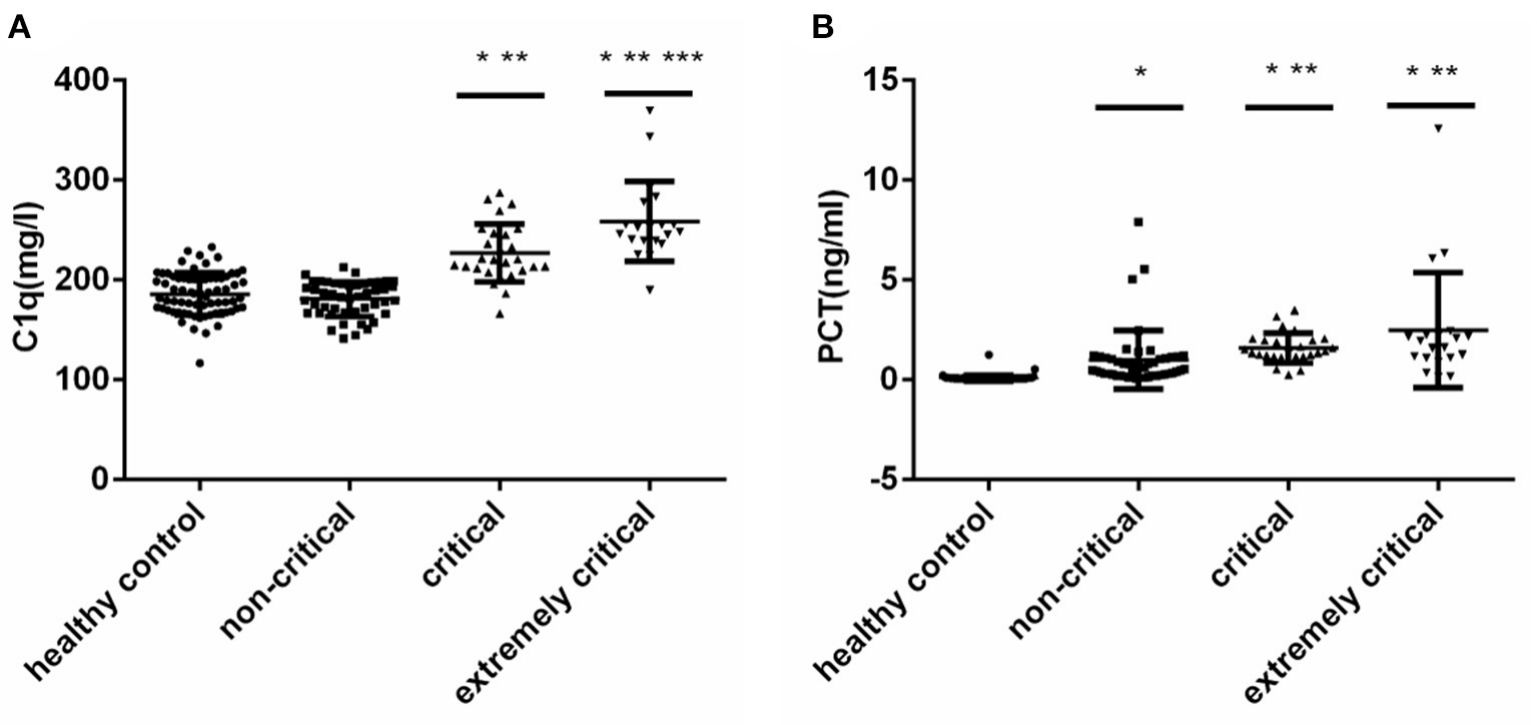
**(A)** Serum C1q levels in different subgroups of children with sepsis. **(B)** Serum PCT levels in different subgroups of children with sepsis. Serum C1q and PCT levels in different subgroups of children with sepsis. The non-Gaussian data C1q and PCT among multiple groups were compared by Kruskal-Wallis *H* test, and then compared in pairs. *: Compared with healthy control group, *P* < 0.05; **: Compared with non-critical group, *P* < 0.05; ***: Compared with critical group, *P* < 0.05.

According to PCIS, children with sepsis were divided into non-critical group, critical group and extremly critical group. And there was no significant difference in age and gender among the four groups (Figure 1) (*P* > 0.05). The results showed that the levels of C1q were 182.80 (166.75, 195.85) mg/L, 219.90 (209.10, 246.40) mg/L and 249.95 (239.10, 272.25) mg/L in non-critical group, critical group and extremely critical group respectively, which were in direct proportion to the severity of the disease (Figure 1A) (*P* < 0.001). Except the non-critical group, the levels of C1q in the other two subgroups were higher than those in the healthy control group. At the same time, Figure 1B illustrated that there was no significant difference in PCT between critical group [1.40(1.23, 1.94) ng/ml] and extremely critical group [1.77(1.08, 2.23) ng/ml], but the levels of PCT in non-critical group [0.52(0.21, 1.12) ng/ml] were lower than that in critical and extremely critical group (*P* < 0.001). The PCT levels of the three subgroups were all higher than those of the healthy control group.

### Correlation Analysis of Serum C1q and PCT in Children With Sepsis

**Figure 2:**
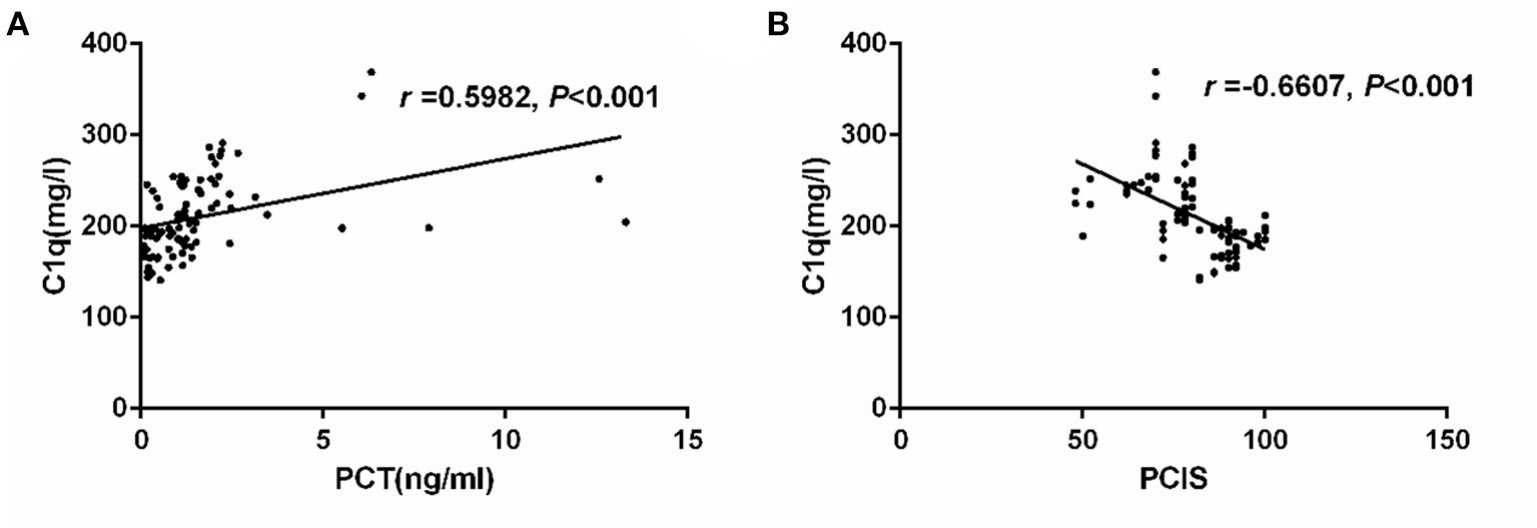
**(A)** Correlation of serum C1q with PCT in children with sepsis. **(B)** Correlation of serum C1q with and PCIS in children with sepsis. Correlation of serum C1q with PCT and PCIS in children with sepsis.

We conducted Spearman correlation analysis and found that there was a significant positive correlation between serum C1q and PCT levels in children with sepsis (*r* = 0.5982, *P* < 0.001) (Figure 2A). Furthermore, we also analyzed the correlation between C1q and PCIS levels, and the results showed that there was also a significant correlation between the two, and the correlation coefficient is −0.6607 (*P* < 0.001) (Figure 2B).

### Serum C1q Levels in Children With Sepsis Infected by Different Pathogens

**Figure 3:**
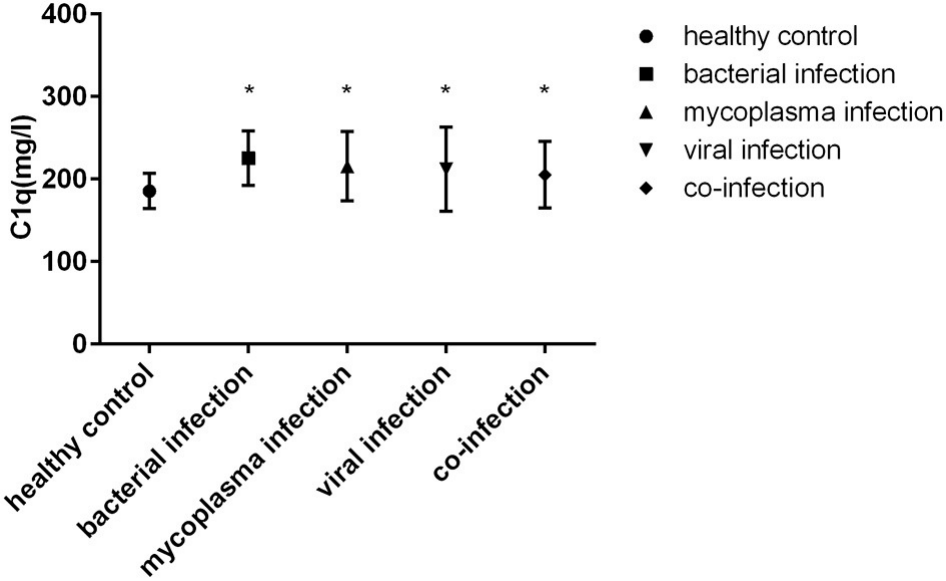
Serum C1q levels in children with sepsis infected by different pathogens. *: Compared with healthy control group, *P* < 0.05.

The serum C1q levels of children with four different pathogens (bacterial infection, mycoplasma infection, viral infection and co-infection) were higher than those of healthy control group (*P* < 0.05). However, there was no significant difference in serum C1q levels among children infected with different pathogens (Figure 3).

## Discussion

With the improvement of medical conditions, the incidence and mortality of sepsis have declined in recent years, but it is still the main cause of global health damage (4). Early diagnosis and timely response can significantly improve the prognosis of children with sepsis. This article included 166 children as the research object. Because the functions of the children's body systems, especially the immune system, are still not complete, they are vulnerable to the invasion of pathogens from the outside, and the body's response to pathogens varies with age after infection, at the same time, the reserve of organ function of children is poor, which leads to the obvious difference between children's clinical manifestations and that of adult cases, the scoring system based on adults cannot be directly applied to children. The First Aid Group of the Pediatrics Branch of the Chinese Medical Association revised the PCIS in 1995, the more serious the disease, the lower the PCIS score. The results of our study showed that the serum C1q levels of children with sepsis were higher than that of healthy children and the C1q levels of children in different subgroups were distinct. The serum C1q levels was negatively correlated with the PCIS so we speculated that C1q was positively correlated with the severity of the disease. This suggests that C1q may also be a serum marker of children with sepsis and can evaluate the severity of the disease.

Serum complement C1q has not been studied in children with sepsis. As one of the important components of complement C1, complement C1q has the largest molecular structure with a relative molecular mass of about 410,000 (21). It is a glycoprotein composed of 18 polypeptide chains. Unlike most other complement proteins that are mainly derived from the liver, complement C1q is mainly synthesized by macrophages (23). Complement is an important part of human immunity (24). Serum complement C1q together with C2 and C4 participates in the activation of the classical pathway of complement, and eventually form C3 convertase (C4bC2a). Serum complement C1q is the initial part of the classical complement pathway (25). Studies have shown that serum C1q levels are distinct in different diseases. For example, serum C1q levels are elevated in patients with atherosclerosis (26) and acute ischemic stroke (27). However, it is reduced in the serum of patients with multiple myeloma (28), but there is no relevant research to confirm the value of serum complement C1q in the evaluation of sepsis. The results of this study showed that serum C1q in children with sepsis was increased, and it was related to the severity of the disease. The more serious the disease was, the higher the serum C1q levels were. As we all know, under physiological conditions, the activation and regulation of the complement system are in a state of equilibrium, which protects the body from foreign bodies. When the balance is broken, the function of the complement system will be disordered and may attack its own cells or tissues, leading to a variety of inflammatory reactions and organ damage (29). C1q is an important initiation molecule of the classical activation pathway of the complement system. When the body is infected by pathogens and causes sepsis, C1q can recognize the complement binding site of antibody Fc segment in IgG or IgM immune complex, activate the complement cascade reaction, and clear antigen-antibody complex (30). In this study, it was observed that the serum C1q level of children with sepsis increased, which may also be due to the body's adjustment of the balance of the complement system, compensating to produce more C1q to resist severe inflammatory response, with the development of disease and the aggravation of inflammatory response, the level of C1q gradually increased; On the other hand, the increased level of C1q may also be an immune regulation of the body in the initial stage of sepsis. Studies have found that C1q can recognize apoptotic cells (31). We speculate that apoptosis after cell injury during sepsis would be recognized by C1q, and then quickly initiate cell phagocytosis through the complement-dependent immune regulation pathway, clear apoptotic cells, inhibit the inflammatory response of sepsis, and help the body maintain immune tolerance (32).

It is known to all that the most common biomarker applied to sepsis is PCT both in human and animals (33–35). The level of PCT in normal organism is very low (<0.1 ng/ml). When the body is infected, the PCT level will rise rapidly, and the serum PCT levels can be significantly increased in the early stage of sepsis. The PCT levels of children with sepsis in this study were significantly higher than that of healthy children, which is the same as that of previous studies (36). However, although the non-critical group was lower than the critical group and the extremely critical group, there was no difference between the critical group and the extremely critical group. This also means that PCT has certain predictive significance for the severity of sepsis, but it cannot distinguish between critical group and extremely critical group, which is different from previous studies (36), we consider that it may be due to the sample size, we will expand the sample size for further analysis. Furthermore, we also found that there was a significant positive correlation between serum C1q levels and PCT levels which suggested that both C1q and PCT were involved in the occurrence and development of sepsis, and could reflect the severity of sepsis. This further indicates that serum C1q level has a certain reference value for the preliminary diagnosis and severity classification of sepsis.

## Conclusion

In summary, our study found that the serum C1q levels of children with sepsis were elevated, and it was negatively correlated with the PCIS score, which can be used as an indicator to assess the severity of the disease in children with sepsis. At the same time, the study found that there was a positive correlation between C1q levels and PCT levels in children with sepsis, which indicated that C1q indeed participated in the occurrence and development of sepsis. At present, there are few studies on C1q in sepsis. We suggest that scholars can expand the sample size for more in-depth research to clarify the specific path and mechanism of C1q in children with sepsis.

## Data Availability Statement

The raw data supporting the conclusions of this article will be made available by the authors, without undue reservation.

## Ethics Statement

Ethical review and approval was not required for the study on human participants in accordance with the local legislation and institutional requirements. Written informed consent to participate in this study was provided by the participants' legal guardian/next of kin.

## Author Contributions

HL completed the data processing and manuscript writing. PZ was responsible for the revision and review of the paper. JC provided the ideas of the manuscript. YH and XC were responsible for collecting data. All authors contributed to the article and approved the submitted version.

## Conflict of Interest

The authors declare that the research was conducted in the absence of any commercial or financial relationships that could be construed as a potential conflict of interest.
